# ‘Family members screaming for help makes it very difficult to don PPE’. A qualitative study on UK ambulance staff experiences of infection prevention and control practices during the COVID-19 pandemic

**DOI:** 10.1177/17571774231209494

**Published:** 2024-01-08

**Authors:** Peter Eaton-Williams, Julia Williams

**Affiliations:** 1 Senior Research Paramedic, 53259 South East Coast Ambulance Service NHS Foundation Trust, Crawley, UK; 2 Professor of Paramedic Science, School of Health & Social Work, 3769 University of Hertfordshire, Hatfield, UK; 3 Head of Research, College of Paramedics, Bridgwater, UK

**Keywords:** infection prevention and control, personal protective equipment, ambulance, COVID-19, survey, qualitative

## Abstract

**Background:**

During the first wave of the COVID-19 pandemic in the UK, ambulance staff continued to deliver direct patient care whilst simultaneously adapting to a considerable escalation in evolving infection prevention and control (IPC) practices.

**Aim:**

To enable learning to benefit future planning, this qualitative article aims to describe ambulance staff’s experiences of this rapid escalation of IPC practices.

**Method:**

Three online surveys were presented during the acceleration, peak, and deceleration phases of the pandemic’s first wave in the UK (2020). Overall, 18 questions contributed 14,237 free text responses that were examined using inductive thematic analysis at both descriptive and interpretive levels.

**Findings:**

Many participants lacked confidence in policies related to the use of personal protective equipment (PPE) because of perceived inadequate supporting evidence, confusing communication, and low familiarity with items. Compliance with policy and confidence in PPE use were further influenced by discomfort, urgency, and perceptions of risk. Various suggestions were made to improve IPC practices within the work environment, including reducing unnecessary exposure through public education and remote triage improvements.

**Discussion:**

Some participants’ poor experiences of escalating IPC practices were shared with health care workers studied in other environments and in previous epidemics, emphasising the need for lessons to be learnt. PPE should be developed with consideration of ambulance staff’s unique working environment and regular familiarisation training could be beneficial. Pragmatic, evidence-based, clearly communicated policies implemented with sufficient resources may protect staff and facilitate them to maintain standards of care delivery during a pandemic.

## Introduction

In March 2020, following the outbreak of the COVID-19 pandemic, infection prevention and control (IPC) guidance and supplies of personal protective equipment (PPE) began to be disseminated and distributed to health care workers (HCWs) delivering frontline care in the UK (National Audit Office, 2020). Throughout the next few months, NHS ambulance service staff continued to deliver both remote and face-to-face patient care whilst adapting their working practices to include evolving IPC guidance and whilst utilising items of PPE that were previously not in routine use (Thomas et al., 2020; NHS England, 2022). Pandemics represent significant health risks for HCWs, and all efforts must be made to reduce these in order to preserve this critical workforce (Billings et al., 2021). Consequently, in April 2020, the College of Paramedics, in association with the National Ambulance Research Steering Group (NARSG), initiated the COVID-19 Ambulance Response Assessment (CARA) study to enable learning from ambulance staff’s experiences. One particular aim of CARA was to assess ambulance staff’s feelings of preparedness and confidence to deliver patient care during that first wave of the pandemic in the UK.

## Methods

CARA was a Health Research Authority-approved, prospective three-part longitudinal online survey of UK ambulance staff working in patient facing roles, both face-to-face and virtually, and also of registered paramedics working in other frontline environments. Invitations were disseminated by NHS ambulance trusts and the College of Paramedics, and presentations of the survey were timed to coincide approximately with the acceleration, peak, and deceleration phases of the first COVID-19 wave: 02 - 15/04/2020, 02–12/05/2020, and 21/09–12/10/2020, respectively (Office for National Statistics, 2021). Only participants in CARA1 were invited to participate in subsequent surveys, resulting in participant numbers of 3,717 for CARA1; 2,709 for CARA2; and 2,159 for CARA3. Of those initial participants, 82% (*n*=3,055) declared a role involving face-to-face patient care and at least 65% (*n*=2,399) were registered paramedics.

Predominantly, the questions within CARA’s surveys collected quantitative data, but some free text questions collected data most suitable for qualitative exploration as survey participants were able to describe their experiences of the pandemic in their own words. In total, 14,237 responses were collected using 18 different questions (see Appendix 1), enabling a great diversity of thoughts and feelings to be expressed. Inductive thematic analysis (Braun and Clarke, 2006) was applied to data by PEW using NVivo software (2020, QSR International). Individual questions were coded independently of one another, and individual responses were coded to multiple codes where appropriate. All codes and themes identified were concurrently reviewed by JW to benefit credibility (Nowell et al., 2017). Initial descriptive coding was subsequently augmented with the identification of interpretive sub-themes, and these were combined with descriptive sub-themes. This approach allowed us to report participants’ practical needs and underlying emotions simultaneously (Braun and Clarke, 2014). To benefit reflexivity, we declare that PEW is a research paramedic with over 20 years’ experience of ambulance-based clinical practice, whilst JW, also a paramedic, is both a Professor and Head of Research in the field of paramedic science with extensive experience of research in health care, teaching, and clinical practice.

One of the main themes identified during qualitative analysis was an overriding goal expressed by CARA participants to reduce the risk of COVID-19 infection to staff, patients, and loved ones through effective IPC practices. By focussing on this theme alone, this article aims to provide critical insight into ambulance staff’s experiences of IPC practices and PPE use during the COVID-19 pandemic.

### Findings

The IPC theme contained four descriptive sub-themes, all strongly demonstrating the interpretative sub-theme of fear of COVID-19 infection and transmission affecting participants, their colleagues, their loved ones, or their patients:

• PPE policy, communication, and training.
• PPE supply, standard, and fit-testing.
• PPE in use, compliance, and fatigue.
• Other IPC practices.

### PPE policy, communication, and training

Perceptions of insufficient evidence supporting PPE effectiveness and disagreement related to the definition of aerosol-generating procedures (AGPs) caused much confusion, anxiety, and loss of confidence amongst participants.

*‘The PPE is not fit for purpose and despite being allegedly evidence based there seems to be very little evidence to support it’,* C1.Q27a.1129.

*‘Ongoing conflicting viewpoints on CPR* [cardiopulmonary resuscitation] *as an AGP increasing stress’,* C1.Q28a.2605.

Though ambulance services had consistent policies, perceived variations in practice across ambulance services and between receiving hospitals led to frustration, and many participants complained that since the onset of the pandemic PPE guidance had changed frequently, and that those changes were often contradictory which resulted in confusion.

*‘With each visit to ED* [Emergency Department] the *requirement has changed, there is a lot of cross contamination and no one seems to know what they want ambulance crews to do’,* C1.Q45.2864.

*‘Have recently received several conflicting PPE doffing procedures – some safer than others which has become a little confusing’,* C2.Q16a.2210.

To address this confusion and to gain more familiarity with equipment, practical simulation training was advocated by many, and its absence lamented by more. Participants wanted to ensure that donning and doffing procedures were performed correctly and identified cardiac arrest patient management whilst wearing PPE as an area particularly requiring some experience.

*‘First time you do it is for real’,* C2.Q17a.0130.

*‘*[What support is needed?] *Practical simulation of donning/doffing and providing clinical care to patients whilst wearing PPE’, C1Q45.2211.*

Contrastingly, other participants believed that the escalation of PPE use within clinical practice required little explanation or preparation or that they had received adequate quantities of both.

*‘The training provided has been more than adequate’,* C1.Q27a.2512.

‘*The evidence base, training and guidance is out there, it is accessible and disseminated by the trust I work for, however not all colleagues will avail themselves of the opportunity to educate themselves in its use’,* C1.Q28a.2291.

### PPE supply, standard, and fit-testing

Some participants perceived an inadequate supply of PPE, both to Trusts as organizations and in the subsequent distribution to individual stations or crews though this sub-theme diminished as CARA progressed.

*‘I’m sick of stressing about whether we will have enough PPE to last us the whole shift. I want to focus on my role not supplies!’* C1.Q46.0712.

Congruent to doubts expressed about the evidence base supporting PPE effectiveness, were statements indicating perceptions that PPE supplied was of poor quality and inappropriate for use in participants’ working environments. Comments related to the inadequacy of Level 2 (L2) aprons were profuse, with frequent expressions of preference for full body protection, such as surgical gowns, Tyvek suits, or coveralls. L2 surgical masks were similarly maligned, and many called for Level 3 (L3) masks, respirator hoods, eye protection, and/or face visors to be more readily available or accepted policy. Additionally, some participants complained of PPE supplied being out of date or manufactured poorly.

*‘The aprons provided are sub-standard; they do not cover our uniform, our arms are still exposed and when taking patients outside, the aprons fly up in the wind and contaminate us further’,* C1.Q45.0460.

*‘I do not feel that a surgical mask is adequate protection’,* C3.Q28b.0647.

*‘FFP3 masks 6 years past their use by date’,* C2.Q16a.0210.

The importance of timely fit-testing was stressed as were the coexistent needs to subsequently supply items consistent to those tested upon and to provide appropriate alternatives when participants failed. Some participants proposed that personal issue of reusable items would be the most effective strategy to maintain availability of effective PPE.

*‘Got fit tested for one mask but supplies keep changing’,* C2.Q16a.2347.

*‘Failed fit testing for mask and told hood required but no hood provided’,* C1.Q27a.2150.

‘*Personal issue level 3 would be better*’, C3.Q18a.2048.

### PPE in use, compliance, and fatigue

Practical difficulties encountered when using PPE were expressed repeatedly. Restrictions to movement, vision, and communication were commonly described.

*‘Communication is very difficult and the Tyvek suit can be restrictive’,* C1.Q28a.1106.

*‘It’s too hot, cumbersome, noisy and poor vision to run and manage a cardiac arrest in PPE’,* C3.Q19a.2032.

The time required to don PPE, particularly L3, was a varied concern. There were significant moral and emotional pressures encountered when attending time-critical patients, both from the presence of relatives at scene and from participants’ desires to initiate care. There were others concerned that delays to interventions severely reduced chances of a successful outcome.

‘*Family members screaming for help makes it very difficult to don PPE’,* C1.Q28a.1601.

*‘My priorities are a good outcome for the patient. If donning PPE causes unnecessary delay I will adapt on the job’,* C3.Q19a.1811.

*‘Personally given the pre-COVID survival rates I don’t think we should be attempting CPR beyond shockable rhythms as chances of survival is slim, the added PPE etc and delay with limited ITU resources means most attempts will be futile’,* C1.Q28a.0501.

In CARA3, one question introduced the subject of ‘PPE fatigue’ (see Appendix 1, C3Q16), suggesting that some clinicians did not always comply to PPE guidelines due to the tiring nature of its use. Participants were asked to identify factors that influenced their decisions of what PPE to wear. Factors identified included peer influence, weather conditions, the environment, the type of patient, and the nature of their complaint.

*‘What my fellow colleagues are wearing and talking about wearing. Peer pressure’,* C3.Q16a.1160.

*‘Less likely to be fully compliant in hot weather’,* C3.Q16a.1028.

*‘Often not indicated, often impairs communication with elderly patients. Often causes added inhibitions with young patients’,* C3.Q16a.0100.

Lastly, responses to many questions related to PPE revealed that some participants felt that they should have autonomy to decide appropriate levels of use following dynamic risk assessments of the incidents that they are attending.

*‘Although there appears to be conflicting advice on which PPE to wear for cardiac arrest, I will risk assess and use what I feel is safest for me even if my trust states this is not required’,* C2.Q17a.0264.

*‘Allow dynamic risk assessments when donning aprons and goggles. If no aspirates / aerosols are being generated in a level 2 based environment, why wear the apron / goggles?’* C3.Q17a.2038.

### Other IPC practices

Throughout all stages of CARA, the need to improve the cleanliness of work environments and to enable social distancing within them was reiterated.

*‘Proper distancing, we are within 2/3 feet of each other – Less when we are mentoring new staff and we are told it’s ok as long as we are beside each other and not face to face’,* C2.Q43.0947.

Colleague teams were suggested to reduce the numbers of close contacts, and participants also called for regular testing of staff and for feedback on patients encountered who were later confirmed to be COVID-19 positive.

*‘Keep people in bubbles, not change crew members every single shift’,* C3.Q46.1515.

*‘Tests for frontline staff and updates on suspected patients’,* C1.Q45.0202.

Facilitating appropriate remote working practices and providing alternative accommodation for staff with vulnerable household members were both requests and acknowledgements.

Vehicle decontamination between incidents was another frequently addressed topic with many suggesting that dedicated personnel should perform this task, permitting clinicians to rest or complete other tasks. Others underlined that if clinicians did decontaminate vehicles, then they should receive additional training and adequate time to complete this task must be allocated to them.

*‘Have make ready* [vehicle preparation personnel] *at the hospital to assist with preparing vehicles’,* C3.Q46.1663.

Finally, public education, effective triage, and good communication during dispatch were all suggestions to reduce clinician exposure to potential COVID-19 carriers.

*‘Follow the example of other HCP* [Health Care Professional] *groups and limit the unnecessary exposure we have to patients by doing more phone consultations, re-triaging 111 calls, using other care pathways more’,* C3.Q46.1150.

## Discussion

Limitations of our analysis include that all questions were coded independently of each other, meaning we were unable to link responses submitted by individual participants. We did not explore variations across different trusts or settings either, and findings are presented at a national level only. It should also be remembered that due to the number and diversity of responses submitted, it is not possible to represent all participants’ views within our analysis and additionally, the survey method does not permit clarification or further enquiry into statements where this might be of benefit, leading to possible misunderstanding and misrepresentation (Braun et al., 2020). However, there is little doubt that perceptions of discordance on PPE guidance and AGP definition amongst national and international organisations relevant to ambulance staff caused significant distress and distrust. Rapid updates to guidance and variation in procedures across different clinical environments added to confusion. Unfortunately, these are familiar complaints for health care workers, common to previous epidemics of highly infectious diseases (Billings et al., 2021; Houghton et al., 2020). These syntheses of previous qualitative studies identified that ways to combat low confidence in PPE were to provide adequate training and demonstrate evidence of effectiveness, factors regrettably identified again by many participants in our study. The introduction of social distancing measures creates significant barriers to providing supplementary face-to-face training, and perhaps familiarity with PPE use would benefit from an increased emphasis on IPC practices within annual mandatory sessions (Health Education England, 2020). Further research is certainly required on both PPE efficacy and AGP definition (Brown and Chan, 2020; Couper et al., 2020; Jackson et al., 2020; Public Health England, 2021; Verbeek et al., 2020).

Participants particularly doubted the efficacy of L2 PPE, with many advocating higher levels of protection than outlined by guidance (Public Health England, 2021), a view supported by a literature review at the time (Thomas et al., 2020). Nationally, the initial stock, subsequent acquisition, and distribution of PPE were inadequate (National Audit Office, 2020), and participants shared some local experiences of this. Many also highlighted difficulties their employers had adhering to guidance related to fit-testing L3 masks (Public Health England, 2021). Namely, that it must be timely, relevant to the model supplied, and alternative PPE provision or removal from exposure must follow a failed test.

Compounding perceptions of insufficient evidence of efficacy were perceptions of poor quality and inappropriateness of the PPE supplied. Ambulance-based clinical practice is performed in a unique environment, distinct from other frontline HCWs. It includes all varieties of indoor and outdoor locations, in addition to both ambulance and hospital clinical settings (College of Paramedics, 2018). Discomfort and practical difficulties associated with use are shared with all HCWs (Houghton et al., 2020; Parush et al., 2020), but consideration of this distinction should surely influence what items of PPE are supplied to ambulance personnel.

Another consequence of ambulance-based practice was the moral dilemma experienced between providing emergency care and taking appropriate self-protection precautions, demonstrated by this article’s title. Participants calling for autonomy to use dynamic risk assessments to determine the level of PPE required received support from both the College of Paramedics (2021) and the Association of Ambulance Chief Executives (2021). This support focused upon escalation of PPE level. It is perhaps equally important that autonomy of IPC practice must not permit emotional pressures to endanger staff via PPE omission.

Issues related to the time taken to don PPE and also to the motor and sensory restrictions identified during task performance might benefit both from targeted design of PPE for ambulance staff and again from increasing their familiarity with those items via more regular face-to-face training (Parush et al., 2020; Verbeek et al., 2020). Indeed, participants confirmed that comfort, perceived benefit, and familiarity would all enhance compliance and diminish fatigue associated with PPE use.

Both donning time and physical restrictions, together with COVID-19 pathophysiology and hospital capacity, were felt by some participants to necessitate some re-evaluation of out-of-hospital cardiac arrest (OOHCA) management. An increased incidence of OOHCA during peak periods of pandemic outbreak, coupled with significantly lower survivability rates (Fothergill et al., 2021; Lai et al., 2020), would seem to support these calls for a degree of pragmatism.

Participants called for better adherence to national IPC guidance and suggested other ways to improve IPC practices at work. Many were frustrated when extended use of PPE was mandated by employers but other strategies, such as contact tracing, had not similarly been implemented. The prolonged delay to enabling staff testing (Health and Social Care Select Committee, 2020) was another significant source of frustration. Calls to improve public education and triage processes to reduce unnecessary clinician exposure in CARA1 and CARA2 coincided with a significant drop in the rate of transportation to hospital (NHS England, 2022). Ambulance clinicians were physically attending over 10% more patients than normal who then subsequently either declined or did not require further hospital assessment or treatment. Whilst ‘hear and treat’ figures (patients receiving care advice solely by phone) showed a smaller corresponding rise (NHS England, 2022), the continued development of these alternative remote care pathways, including the use of video consultations, would thus seem to be merited and they remain priorities within ambulance service strategy (Association of Ambulance Chief Executives, 2020; Health and Social Care Select Committee 2020).

## Conclusions

It is imperative that the experiences of UK ambulance staff during the initial outbreak of the COVID-19 pandemic are heeded and considered by leadership when planning to ensure quality health care delivery in potential future pandemics of infectious pathogens. This study demonstrates that ambulance staff shared many experiences and frustrations related to escalating IPC practices with HCWs working in a variety of clinical environments studied during this pandemic. Disappointingly, it also shows that many of these were shared by HCWs studied in previous epidemics, reinforcing the requirement to learn from this pandemic’s experiences.

This study highlights that there are also differences between ambulance staff and other health care professional colleagues, particularly related to the emergency nature of some incidents and an uncontrolled prehospital environment. PPE designed with these distinctions in mind, coupled with more regular face-to-face training to ensure familiarity with their use, is likely to improve both performance and acceptance related to PPE use in the future. Participants in our study underlined the importance of pragmatic IPC policies related to their occupational environment and their clinical practice, that prioritise their personal safety and have demonstrable evidence supporting them. Guidance relating to changing IPC policies must be communicated effectively with sufficient resources provided to enable their successful implementation.

## Appendix 1: CARA questions used in qualitative data collection.

(Questions most represented in this article are shaded.)

**Table:**
